# Loss of *famh-136/* FAM136A results in minor locomotion and behavioral changes in *Caenorhabditis elegans*

**DOI:** 10.17912/micropub.biology.000553

**Published:** 2022-06-02

**Authors:** Chieh-Hsiang Tan, Heenam Park, Paul W. Sternberg

**Affiliations:** 1 Division of Biology and Biological Engineering, California Institute of Technology, Pasadena, CA 91125, USA

## Abstract

Not much is known about FAM136A, a human gene that may be involved in Meniere’s disease and is conserved throughout animals. To understand the function of *famh-136* , the *Caenorhabditis elegans* ortholog of FAM136A, loss of function alleles of the gene were generated. We find that loss of *famh-136* function results in minor but significant changes to the locomotion and behavior.

**Figure 1. Loss of famh-136/ FAM136A results in slightly larger worms with minor locomotion and behavioral changes.**
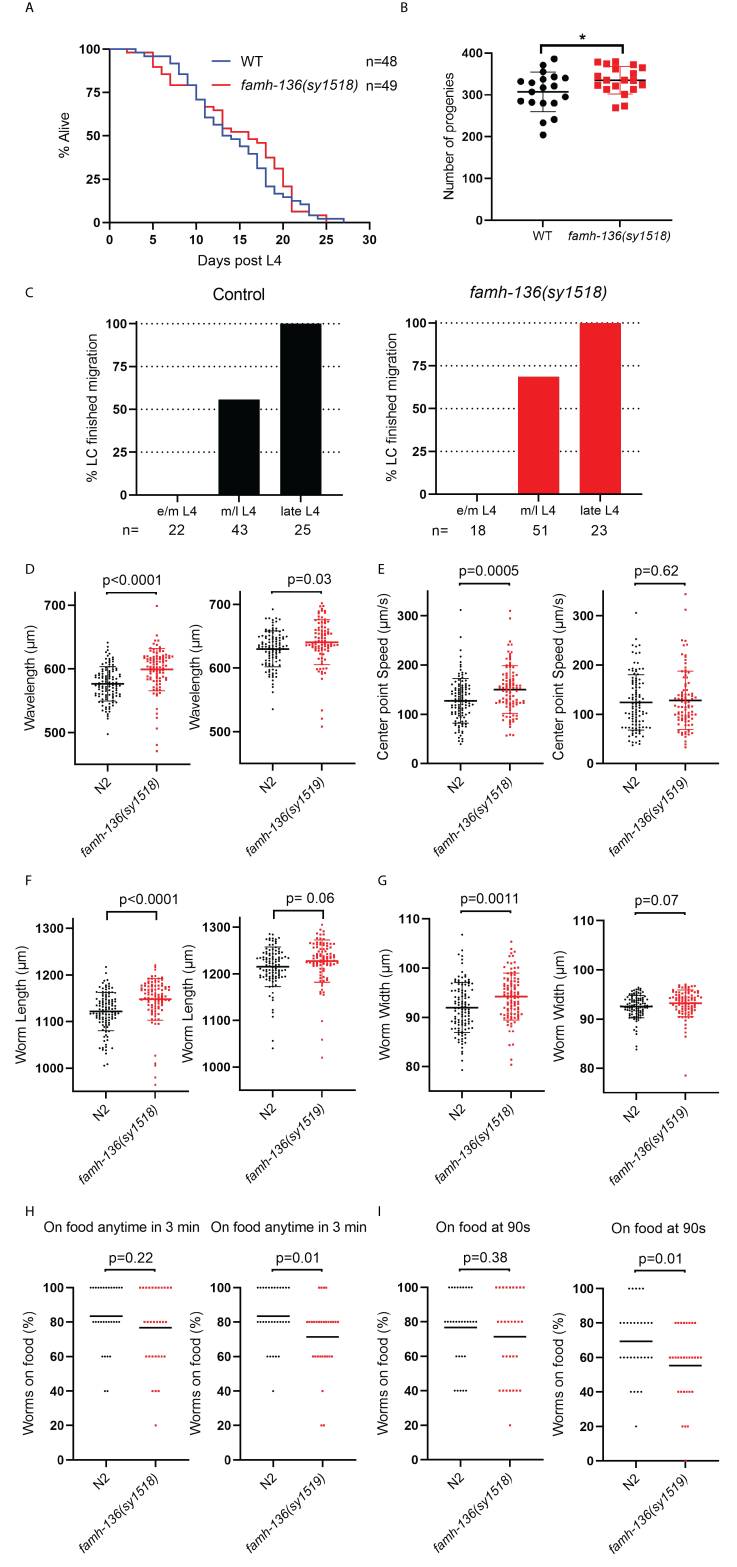
A) *famh-136(sy1518)* animals have a similar life span compared to that of the wild type. P=0.52 (Log-rank test). B) *famh-136(sy1518)* animals have a slightly larger brood size compared to that of the wild type (n= 19 for each genotype). * *P* <0.05 (Student’s *t* -test). C) Linker cell migration is not affected by *famh-136(sy1518)* mutation. Right: *syIs128; him-5(e1490)* control; left: *syIs128; famh-136(sy1518); him-5(e1490)* . e/m L4: early/ mid-L4; m/l L4: mid/late-L4. D-E) *famh-136* mutant animals move slightly differently compared to that of the wild-type. D) Both *famh-136(sy1518)* and *famh-136(sy1519)* have a slightly but significantly longer wavelength (Student’s *t* -test). E) Both *famh-136(sy1518)* and *famh-136(sy1519)* move slightly faster, but it is only significantly so with *famh-136(sy1518)* (Student’s t-test). F-G) *famh-136* mutant animals were slightly larger in than the wild type. Measurement of both the animal length and width shows that *famh-136(sy1518)* and *famh-136(sy1519)* were slightly larger, but it is only significantly so with *famh-136(sy1518)* (Student’s t-test). H-I) *famh-136* mutant animals were less likely to stay on OP50 food lawns H) *famh-136* mutant animals were less likely to stay on OP50 food lawns at any moment in a 3 min time period. The difference is only significant with *famh-136(sy1519).* I) *famh-136* mutant animals were less likely to be on OP50 food lawns at the midpoint (90s) of the 3 min time period. The difference is only significant with *famh-136(sy1519).*

## Description

The human gene FAM136A (family with sequence similarity 136 member A) has been associated with familial Meniere’s disease (MD) (Requena et al., 2015), a chronic disorder of the inner ear (Nakashima et al., 2016). There is also a suggestion that the expression of the gene could be associated with lung cancer prognosis (Zhao et al., 2020). FAM136A is evolutionary conserved, with orthologs identified in various metazoan species (Alliance of Genome Resources, 2022). However, with the exception of some expression data and omics level analysis (Alliance of Genome Resources, 2022; Stuckenholz et al., 2009), very little is known about FAM136A.

The *Caenorhabditis elegans* ortholog of FAM136A is *ZK637.2* (Davis et al., 2022), which we rename *famh-136* ( FAM ily with sequence similarity genes H omolog). To elucidate the biological role of *famh-136* / FAM136A, we generated *famh-136* knock-out animals with CRISPR/Cas9, using the STOP-IN cassette strategy as previously described (Wang et al., 2018). Since the STOP-IN cassette introduced an 8 ectopic amino acid and a premature stop after the 28 ^th^ amino acid in the 143 amino acid protein, we predicted that the resulting two independently isolated, but identical knock-out alleles ( *sy1518* and *sy1519* ) are likely to be null. The *famh-136(sy1518)* knock-out animals were superficially wild-type with a similar lifespan (Fig. 1A) and a slightly larger brood size (Fig. 1B).

*famh-136* is predicted to be co-transcribed along with three other genes in the operon CEOP3552 (Davis et al., 2022). *famh-136* is followed by *lnkn-1* , which is expressed in the male linker cell and is required for the normal migration of the male gonad (Kato et al., 2014). However, CEOP3552 is likely a “hybrid operon”, and *lnkn-1* is thought to have an internal promoter (Huang et al., 2007), so the expression pattern is not necessarily similar. In addition, genes located in the same operon are not necessarily related in their function (Blumenthal and Gleason, 2003) but some are (Huang et al., 1994). We were thus interested in whether *famh-136* could be involved in linker cell migration. With the assistance of *lag-2p::YFP* (Kato and Sternberg, 2009) as a marker for linker cell and *him-5(e1490)* (Hodgkin et al., 1979) to increase male occurrence, we found that loss of *famh-136* does not have a significant effect on the migration of the male linker cell. No significant changes were observed both in the attachment of the linker cell to the rest of the gonad nor the timing of the migration (Fig. 1C).

To further analyze the impact of the *famh-136* mutations, we utilize a quantitative tracking system to measure the movement and coordination of the worms. The measurements reveal slight differences in the movement of *famh-136* mutant worms compared with that of the wild-type. The wavelengths of sinusoidal shape movement were slightly but significantly longer (Fig. 1D), and the worms moved slightly faster (Fig. 1E). These changes may be related to the size of the worm, as *famh-136* mutant worms were also slightly larger in size (Fig. 1F-G). Finally, we found that the mutations in *famh-136* also resulted in a minor change in behavior, with mutant worms being slightly less likely to stay in the OP50 food lawn (Fig. 1H-I). Although the two knock-out alleles- *sy1518* and *sy1519* , have identical nucleotide changes, there were some phenotypic variations in our experiments (Fig. 1D-I). It is possible that some of these differences could be the result of a background mutation. However, the direction of the differences between the mutants and the wild-type is always consistent. Some of the differences could likely be caused by variation between experiments rather than between the alleles.

Overall, we showed that loss of *famh-136/* FAM136A results in slightly larger worms with minor locomotion and behavioral changes.

## Methods

**Nematode strains maintenance and general methods**

All *C. elegans* strains were cultured on Nematode Growth Medium (NGM) dishes with a lawn of *Escherichia coli* strain OP50 at 20°C. The reference strain was the wild-type strain Bristol N2 (Brenner, 1974), from which all the strain was derived. Alleles and transgenes used in this study were: *syIs128* [ *lag-2p::YFP* ] II (Kato and Sternberg, 2009), *famh-136(sy1518)* III (this study), *famh-136(sy1519)* III (this study), *him-5(e1490)* V (Hodgkin et al., 1979).

**Generation of *famh-136* KO alleles *sy1518* and *sy1519***

The Genomic information of *famh-136/ ZK637.2* was facilitated by Wormbase (Davis et al., 2022), a knowledgebase for nematode research. We requested the generation of the *famh-136* KO alleles from the knockout consortium, and Heenam Park generated the alleles as part of the consortium. Two identical *famh-136* mutation alleles *sy1518* and *sy1519* were generated using the universal STOP-IN cassette method as described in Wang et al. (2018). The 43bp knock-in cassette (GGGAAGTTTGTCCAGAGCAGAGGTGACTAAGTGATAAgctagc) is inserted in the first exon between CGATGAGATGATTGACGATTTGGATAAGACCTATT and TGAGGGATATGCAGAAGAGCATGTTTCAGTGCTCAG. The insertion introduced an 8 ectopic amino acid and a premature stop after the 28 ^th^ amino acid in the 143 amino acid protein, and a series of premature stops starting after the 36 ^th^ residue.

**Lifespan assay**

WT (N2) and *famh-136(sy1518)* worms synchronized at L1 and cultured to L4 were placed on NGM dishes either in groups or individually. The worms were transferred to fresh dishes daily until they ceased reproduction or death. The survival of the animal is assayed by whether an animal displays spontaneous movement or by responds to light touching with a platinum wire (Huang et al., 2004). Worms that crawl out of the NGM dishes were excluded from the analysis (2 of 50 in N2, and 1 of 50 in *sy1518* ).

**Fertility assays**

WT (N2) and *famh-136(sy1518)* worms were synchronized at L1 and cultured to L4 as described in the lifespan assay. L4 worms were placed individually on NGM plates and transferred to fresh dishes daily until the cease of reproduction or death. Individual brood size numbers were determined by counting the total number of progenies of each worm during its lifespan. The worms assayed in fertility assays were also included in the lifespan assays. Worms that crawled out of the NGM dishes were excluded from the analysis (1 of 20 in N2, and 1 of 20 in *sy1518* ).

**Male linker cell migration analysis**

To track the developmental migration of the male linker cell, transgene *syIs128* [ *lag-2p::YFP* ] (Kato and Sternberg, 2009), which labels the cytoplasm of the linker cell, is utilized as the marker of the cell; and *him-5 (e1490)* (Hodgkin et al., 1979) mutation that increases nondisjunction of the sex chromosome is used to increase the occurrence of males. Staging of the males follows the criteria set out by Kato and Sternberg (2009). Briefly, early/mid-L4 was defined by a large retraction of the hook with jagged, receding edges; mid/late-L4 was defined by the beginning of the tail retraction; and late-L4 was defined by tail retraction that has receded to the base of the tail taper. The observation was done using a Zeiss Imager Z2 microscope equipped with Apotome 2 and Axiocam 506 mono.

**Movement tracking and worm size measurement**

Movement tracking and worm size measurement was performed similarly to what was described in Wong et al. (2019) with some modifications. Briefly, WT (N2) and *famh-136* mutant worms ( *sy1518* or *sy1519* ) were synchronized at L1 and cultured for around 72 hours. At least an hour prior to the experiment, worms were transferred to NGM plates freshly seeded with 50µl of OP50. For each plate, 5 worms were transferred. The recording and tracking of the worm were performed using a WormLab (MBF Bioscience, Williston, VT) equipment and software. The camera was a Nikon AF Micro 60/2.8D with zoom magnification. Each plate was recorded for 3 minutes. The raw data from the WormLab is then manually processed by matching the tracking data with the recorded video. Only data from worms that were continually tracked for at least 1 minute and of good quality were used in Fig1D-G. For Fig1H, worms were scored manually by watching the 3 minute recordings. Contacting food at any moment in the timeframe count as positive. For Fig1G, worms were manually scored based on their position at 90 seconds into the recording.

## References

[R1] Alliance of Genome Resources Consortium . (2022). Harmonizing model organism data in the Alliance of Genome Resources.. Genetics.

[R2] Blumenthal T, Gleason KS. 2003. Caenorhabditis elegans operons: form and function. Nat Rev Genet 4: 112-20. 10.1038/nrg995 12560808

[R3] Brenner S (1974). The genetics of Caenorhabditis elegans.. Genetics.

[R4] Davis P, Zarowiecki M, Arnaboldi V, Becerra A, Cain S, Chan J, Chen WJ, Cho J, da Veiga Beltrame E, Diamantakis S, Gao S, Grigoriadis D, Grove CA, Harris TW, Kishore R, Le T, Lee RYN, Luypaert M, Müller HM, Nakamura C, Nuin P, Paulini M, Quinton-Tulloch M, Raciti D, Rodgers FH, Russell M, Schindelman G, Singh A, Stickland T, Van Auken K, Wang Q, Williams G, Wright AJ, Yook K, Berriman M, Howe KL, Schedl T, Stein L, Sternberg PW. 2022. WormBase in 2022-data, processes, and tools for analyzing Caenorhabditis elegans. Genetics : . 10.1093/genetics/iyac003 PMC8982018 35134929

[R5] Hodgkin J, Horvitz HR, Brenner S (1979). Nondisjunction Mutants of the Nematode CAENORHABDITIS ELEGANS.. Genetics.

[R6] Huang C, Xiong C, Kornfeld K. 2004. Measurements of age-related changes of physiological processes that predict lifespan of Caenorhabditis elegans. Proc Natl Acad Sci U S A 101: 8084-9. 10.1073/pnas.0400848101 PMC419561 15141086

[R7] Huang LS, Tzou P, Sternberg PW. 1994. The lin-15 locus encodes two negative regulators of Caenorhabditis elegans vulval development. Mol Biol Cell 5: 395-411. 10.1091/mbc.5.4.395 PMC301050 8054684

[R8] Huang P, Pleasance ED, Maydan JS, Hunt-Newbury R, Oi'Neil NJ, Mah A, Baillie DL, Marra MA, Moerman DG, Jones SJ. 2007. Identification and analysis of internal promoters in Caenorhabditis elegans operons. Genome Res 17: 1478-85. 10.1101/gr.6824707 PMC1987351 17712020

[R9] Kato M, Chou TF, Yu CZ, DeModena J, Sternberg PW. 2014. LINKIN, a new transmembrane protein necessary for cell adhesion. Elife 3: e04449. 10.7554/eLife.04449 PMC4275582 25437307

[R10] Kato M, Sternberg PW. 2009. The C. elegans tailless/Tlx homolog nhr-67 regulates a stage-specific program of linker cell migration in male gonadogenesis. Development 136: 3907-15. 10.1242/dev.035477 PMC2778740 19906858

[R11] Nakashima T, Pyykkö I, Arroll MA, Casselbrant ML, Foster CA, Manzoor NF, Megerian CA, Naganawa S, Young YH. 2016. Meniere's disease. Nat Rev Dis Primers 2: 16028. 10.1038/nrdp.2016.28 27170253

[R12] Requena T, Cabrera S, Martín-Sierra C, Price SD, Lysakowski A, Lopez-Escamez JA. 2015. Identification of two novel mutations in FAM136A and DTNA genes in autosomal-dominant familial Meniere's disease. Hum Mol Genet 24: 1119-26. 10.1093/hmg/ddu524 PMC4834881 25305078

[R13] Stuckenholz C, Lu L, Thakur P, Kaminski N, Bahary N. 2009. FACS-assisted microarray profiling implicates novel genes and pathways in zebrafish gastrointestinal tract development. Gastroenterology 137: 1321-32. 10.1053/j.gastro.2009.06.050 PMC2785077 19563808

[R14] Wang H, Park H, Liu J, Sternberg PW (2018). An Efficient Genome Editing Strategy To Generate Putative Null Mutants in
*Caenorhabditis elegans*
Using CRISPR/Cas9.. G3 (Bethesda).

[R15] Wong WR, Brugman KI, Maher S, Oh JY, Howe K, Kato M, Sternberg PW (2019). Autism-associated missense genetic variants impact locomotion and neurodevelopment in Caenorhabditis elegans.. Hum Mol Genet.

[R16] Zhao L, Ge C, Zhang Z, Hu H, Zhang Y, Zhao W, Li R, Zeng B, Song X, Li G. 2020. FAM136A immunoreactivity is associated with nodal involvement and survival in lung adenocarcinoma in a Chinese case series. Bioengineered 11: 261-271. 10.1080/21655979.2020.1735611 PMC7051133 32098576

